# Surrogacy analysis of intermediate end-points for overall survival in randomized controlled trials of rhabdomyosarcoma

**DOI:** 10.1038/s41598-022-23944-w

**Published:** 2022-11-12

**Authors:** Yuta Kubota, Kazuhiro Tanaka, Masanori Kawano, Tatsuya Iwasaki, Ichiro Itonaga, Hiroshi Tsumura

**Affiliations:** Department of Orthopedic Surgery, Faculty of Medicine, Oita University, 1-1 Idaigaoka Hasama, Yufu City, Oita 879-5593 Japan

**Keywords:** Oncology, Cancer, Sarcoma, Cancer, Sarcoma

## Abstract

Treatment of malignant tumors, such as rhabdomyosarcoma (RMS), can improve overall survival (OS). It is time-consuming and expensive for patients to obtain benefits from randomized controlled trials (RCTs) with OS as the primary end-point. Therefore, another surrogate end-point is necessary; however, there is no report on the surrogacy analysis of RMS. In this study, we performed a systematic review of RCTs, involving patients with newly diagnosed RMS, and 11 RCTs were identified. We performed a meta-analysis to assess the surrogacy of intermediate end-points for OS. The correlations between surrogate end-points and OS were investigated using Spearman's rank correlation coefficient (ρ). The coefficient of determination (R^2^) was employed to measure the strength of the association. A total of 5183 patients were randomly allocated to 34 treatment groups. A marginal correlation (R^2^ = 0.281, ρ = 0.445) between the hazard ratios (HRs) for event-free survival (EFS) and OS was observed. In patients with localized RMS, the EFS HR had a weaker correlation with OS HR in the sensitivity analysis than that in the primary analysis. Overall, the surrogacy of EFS for OS cannot be confirmed.

## Introduction

Rhabdomyosarcoma (RMS) is one of the most common soft tissue sarcomas (STS) in children and adolescents. It accounts for 10.3% of STS in Japanese children, and adolescents and young adults (AYA) aged between 15 and 39 years^1^; the incidence rate of RMS is 4 per 100,000 individuals for the 0–19-year age group in Europe^2^. Moreover, RMS in children and AYA accounts for approximately 64% of all RMS cases^1^. In children and adolescents, the two common RMS subtypes are embryonal RMS (ERMS), accounting for approximately 57% of RMS and alveolar RMS (ARMS), accounting for approximately 26% of RMS^3^. Children and AYA with ERMS present a better prognosis than those with ARMS^3^. Furthermore, approximately 66% of ARMS patients with the paired box 3/7-forkhead box O1 (*PAX3/7-FOXO1*) fusion genes are regarded as a high-risk population^4^.

Since the 1970s, the Intergroup Rhabdomyosarcoma Study Group (IRSG) has performed certain clinical trials that elucidated prognostic factors such as histology, tumor location, and presence of metastasis, and a multi-disciplinary treatment comprising chemotherapy, radiotherapy, and surgery, which have led to a better prognosis^5–9^. Based on these results, in the 2000s, some investigational chemotherapy regimens were established for each risk group by the Children's Oncology Group (COG), USA, European pediatric Soft Tissue Sarcoma Study Group (EpSSG), and Japan Rhabdomyosarcoma Study Group (JRSG). Currently, alkylating agents, including cyclophosphamide and ifosfamide, are used in combination with vincristine and dactinomycin, regarded as standard chemotherapy regimens^5–9^. Additionally, camptothecin-11 (CPT-11) is considered to improve chemotherapy efficacy in children and adults with relapsed RMS^10^. Nevertheless, the difference in overall survival (OS) of patients with ARMS and those with intermediate-risk ERMS between the standard and experimental arms in phase III randomized controlled trials (RCTs) is not significant. Moreover, a few effective chemotherapies are available for patients with relapsed and metastatic RMS. Therefore, future RCTs with high-quality evidence should be conducted to find favorable chemotherapy regimens, including drug selection, administration sequence, dosage, and dosing intervals. It is definitive that treating malignant tumors, such as RMS, can improve OS. OS is the clearest end-point in which an exact point in time can be defined. However, if OS is set as the primary end-point, the clinical trial requires a very large population, huge cost, high follow-up rate, and long-term follow-up. OS is affected by the subsequent treatments. Furthermore, even if there is no treatment effect on survival post-progression (SPP), the OS comparison is affected^11^. New treatments and drugs will be introduced in the future owing to medical science developments. In such an era, it would take a long time for children and AYA with malignant tumors to receive benefits from RCTs defining the primary end-point as OS, verifying the effectiveness of the new treatment. Thus, another surrogate end-point must be defined as the primary end-point in RCTs of such diseases. In osteosarcoma occurring in children and AYA, event-free survival (EFS) or pathological response rate (RR) is frequently set as a primary end-point^12^. However, in RMS, EFS or disease-free survival (DFS) is often set as a primary end-point (Table 1). On the contrary, there are no studies on the relationship between such intermediate end-points and OS focusing on the RCTs of RMS. We performed a meta-analysis including all RCTs that involved chemotherapy for newly diagnosed RMS patients and assessed the surrogacy of intermediate end-points for OS (Table 1)^5–9,13–18^.

**Table 1:** Characteristics of included randomized control trials.

Author	Trial	Study phase	Stage or group or risk	Median follow-up, year	Primary end-point	Secondary end-point	Treatment (sample size, no.)			ITT anal-ysis
							Control	Experimental		
Carli et al. (1988)^13^	RMS-79	ND	Group III − IV	2.3–2.4	RR, Toxicity	ND	VAC (33)	VAC-intramuscularly (37)		No
Maurer et al. (1988)^5^	IRS-I	ND	Group I	8	DFS, OS	ND	VAC (43)	VAC + RT (43)		No
			Group II				VA + RT (87)	VAC + RT (90)		
			Group III − IV				VAC + RT (207)	VAC + ADM + RT (202)		
Maurer et al. (1993)^6^	IRS-II	ND	Group I	7.1	DFS, OS	ND	VAC (37)	VA (64)		No
			Group II				VA + RT (45)	VAC + RT (85)		
			Group III − IV				VAC + RT (294)	VAC + ADM + RT (285)		
Crist et al. (1995)^7^	IRS-III	ND	Group II	4.1–4.3	RR, PFS, OS	ND	VA + RT (44)	VA + ADM + RT (51)		No
			Group III − IV				VAC (+ ADM + DTIC) + RT (87)	VAC + ADM + CDDP(+ VP16) + RT (178)	VAC + ADM + CDDP + VP16(+ DTIC) + RT (174)	
Crist et al. (2001)^8^	IRS-IV	ND	Stage I − III	3.9	FFS, OS	ND	VAC + RT (235)	VAI + RT (236)	VIE + RT (222)	No
Breitfeld et al. (2001)^9^	IRS-IV	Randomized Phase 2	Stage IV	3.5	RR, FFS, OS	ND	VAC + VM + RT (69)	VAC + IE + RT (59)		No
Arndt et al. (2009)^14^	D9803	ND	Inter-mediate Risk	4.3	FFS	OS	VAC + RT (264)	VAC/VTC + RT (252)		No
Oberlin et al. (2012)^15^	MMT95	ND	High Risk	8.6	3-year OS	EFS	VAI (224)	VAI + Carboplatin + VP16 + Epirubicin (233)		No
Hawkins et al. (2018)^16^	ARST0531	Phase 3 RCT	Inter-mediate Risk	4.8	EFS	OS	VAC + RT (222)	VAC/V + Irinotecan + RT (226)		No
Bisogno et al. (2018)^17^	EpSSG RMS 2005	Phase 3 RCT	High Risk	5.3	EFS	OS, RR, toxicity	VAI + RT (242)	VAI + ADM + RT (242)		Yes
Bisogno et al. (2019)^18^	EpSSG RMS 2005	Phase 3 RCT	High Risk	5.0	DFS	OS, toxicity	Stop VAI or (VAI + ADM) + RT (186)	Maintenance VAI or (VAI + ADM) + Vinorelbine + CPA + RT (185)		Yes

Abbreviations: A, dactinomycin; ADM, adriamycin; C, cyclophosphamide; CDDP, cisplatin; CPA, cyclophosphamide; DFS, disease-free survival; DTIC, dacarbazine; E, etoposide; EFS, event-free survival; EpSSG, the European paediatric Soft tissue sarcoma Study Group; FFS, failure-free survival; I, ifosfamide; ITT, intention-to-treat; IRS, the intergroup rhabdomyosarcoma study; M, melphalan; MMT, malignant mesenchymal tumor; ND, not described; OS, overall survival; RCT, randomized controlled trial; RMS, rhabdomyosarcoma; RR, response rate; RT, radiotherapy; T, topotecan; V, vincristine; VP16, etoposide.

## Methods

### Study selection

We systematically searched databases including PubMed, EBSCOhost MEDLINE, Scopus, and the Cochrane Central Register of Controlled Trials (CENTRAL) based on the Preferred Reporting Items for Systematic Reviews and Meta-Analyses (PRISMA) statement^19^. We used keywords “rhabdomyosarcoma,” “chemotherapy,” “randomized controlled trial,” and “randomized, clinical trials” to identify RCTs on RMS reported in English between January 1974 and January 2022. This search was conducted by two authors independently (KT and MK).

### Data and outcome

The date of publication, study title, study duration, inclusion criteria, phase of a clinical trial, primary and secondary outcome measures, intervention and treatment used in each arm, sample size, sex, age, histological type, number and proportion of patients with metastasis, application of intention-to-treat (ITT) analysis, post-protocol therapy, pathological response to chemotherapy, adverse events, and survival data were extracted. For OS, EFS, and RR, the median, hazard ratio (HR) with 95% confidence interval (CI), and P-values were recorded whenever available. DFS and failure-free survival (FFS) were regarded as EFS and were analyzed in this study. The definition of EFS in each trial was adopted. Eight trials defined EFS as relapse or death; six trials defined EFS as disease progression, death, or relapse; one trial defined EFS as disease progression, second malignancy, or death; one trial defined EFS as disease progression, relapse, second malignancy, or death; and one trial defined EFS as death, relapse, disease progression, appearance of a new tumor, change to second-line chemotherapy, or the date of last follow-up. The RR was defined as the proportion of responders (complete or partial) among the assessed patients. Complete and partial definitions were followed for each trial.

The EFS and OS rates for 1-, 3-, and 5-year were extracted from the data using the Kaplan–Meier method. In case these data were not mentioned in the report, they were estimated as binary proportions from each Kaplan–Meier plot for EFS or OS. Toxicity was scored based on the Common Terminology Criteria for Adverse Events (CTCAE) v.5.0, and the focus of this study was CTCAE grade 3 or 4. All information was retrieved and confirmed by two authors (K.T. and M.K.), and the differences among them were arbitrated by Y.K. and T.I.

### Statistical analyses

Pooled HRs with 95% CIs for EFS and OS were calculated using a meta-analysis. Additionally, pooled odds ratios (ORs) with the corresponding 95% CIs for EFS and OS at 1, 3, and 5 years were obtained from a meta-analysis. The Mantel–Haenszel method was applied using the review manager software (version 5.4; Nordic Cochrane Center, Cochrane Collaboration, Copenhagen, Denmark). All remaining statistical analyses were performed by applying the Statistical Analysis System (SAS) (version 9.4; SAS Institute, Cary, NC, USA). P-values were obtained from two-sided tests, and results with *P* < 0.05 were considered significant. Cochrane's Q-test and I^2^ statistics were applied to quantify heterogeneity.

As recommended by Schürmann et al. ^20^, we utilized random effects meta-regression to estimate the relationship between surrogate end-points and OS. Spearman's rank correlation coefficient (ρ) between the surrogate end-points and OS was also estimated. This association strength was assessed by the adjusted coefficient of determination (R^2^). We defined squared coefficient values as excellent (> 0.9), very good (0.9 to < 0.75), good (0.75 to < 0.5), moderate (0.5 to < 0.25), and poor (0.25 ≥).

## Results

### Characteristics of the RCTs included in the meta-analysis

**Figure 1:**
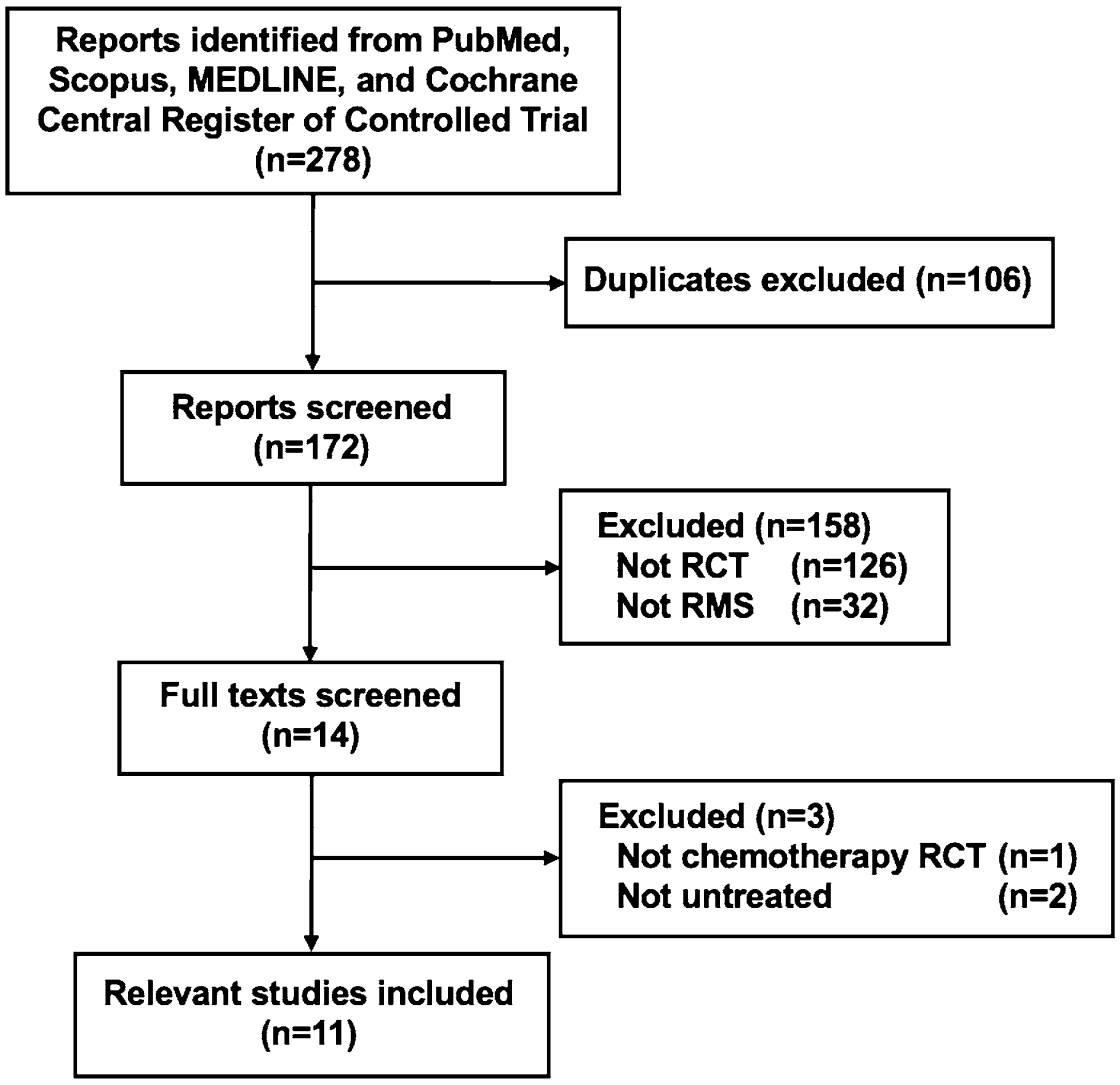
PRISMA flow diagram. Abbreviations: RCT, randomized controlled trial; RMS, rhabdomyosarcoma.

A systematic literature search was conducted, and 278 articles were initially identified for screening. A total of 106 articles were excluded because of duplication, and 172 were screened for eligibility. After screening the abstracts, 158 articles were excluded (not RCT, n = 126; not RMS, n = 32). After 14 full-text screenings, we obtained phase II or III RCTs of chemotherapy that involved chemotherapy-naive patients with newly diagnosed RMS. Ultimately, the remaining 11 RCTs were eligible, and a meta-analysis of such RCTs was performed (Fig. 1). Table 1 shows the main encapsulated features of the included trials. No trial had described the post-protocol treatment in detail.

Collectively, 11 eligible RCTs randomized 5183 patients to 34 treatment arms. Each arm received two to six cytotoxic agents, including vincristine and actinomycin. A total of 27 treatment arms received radiotherapy, and 645 patients (12.4%) diagnosed with metastatic disease were enrolled in seven treatment arms. Neo-adjuvant chemotherapy, adjuvant chemotherapy, and chemotherapy without surgery were administered to 6, 21, and 7 treatment arms, respectively. EFS, DFS, or FFS was adopted to be the primary end-point in four RCTs. Both OS and EFS (or DFS and FFS) were used in five RCTs, and two of them also chose the primary end-point as RR. Another RCT defined the primary end-point as 3-year OS, and another defined the primary end-point as RR and toxicity.

Except one RCT, either EFS or OS was considered the primary or secondary end-point in all RCTs. Histology subtypes included ARMS (n = 1534, 29.6%), ERMS (n = 2794, 53.91%), pleomorphic/other RMS (n = 97, 1.87%), and Ewing/undifferentiated pleomorphic sarcoma (n = 356, 6.87%).

### Meta-analysis of treatment effects and adverse events

**Figure 2:**
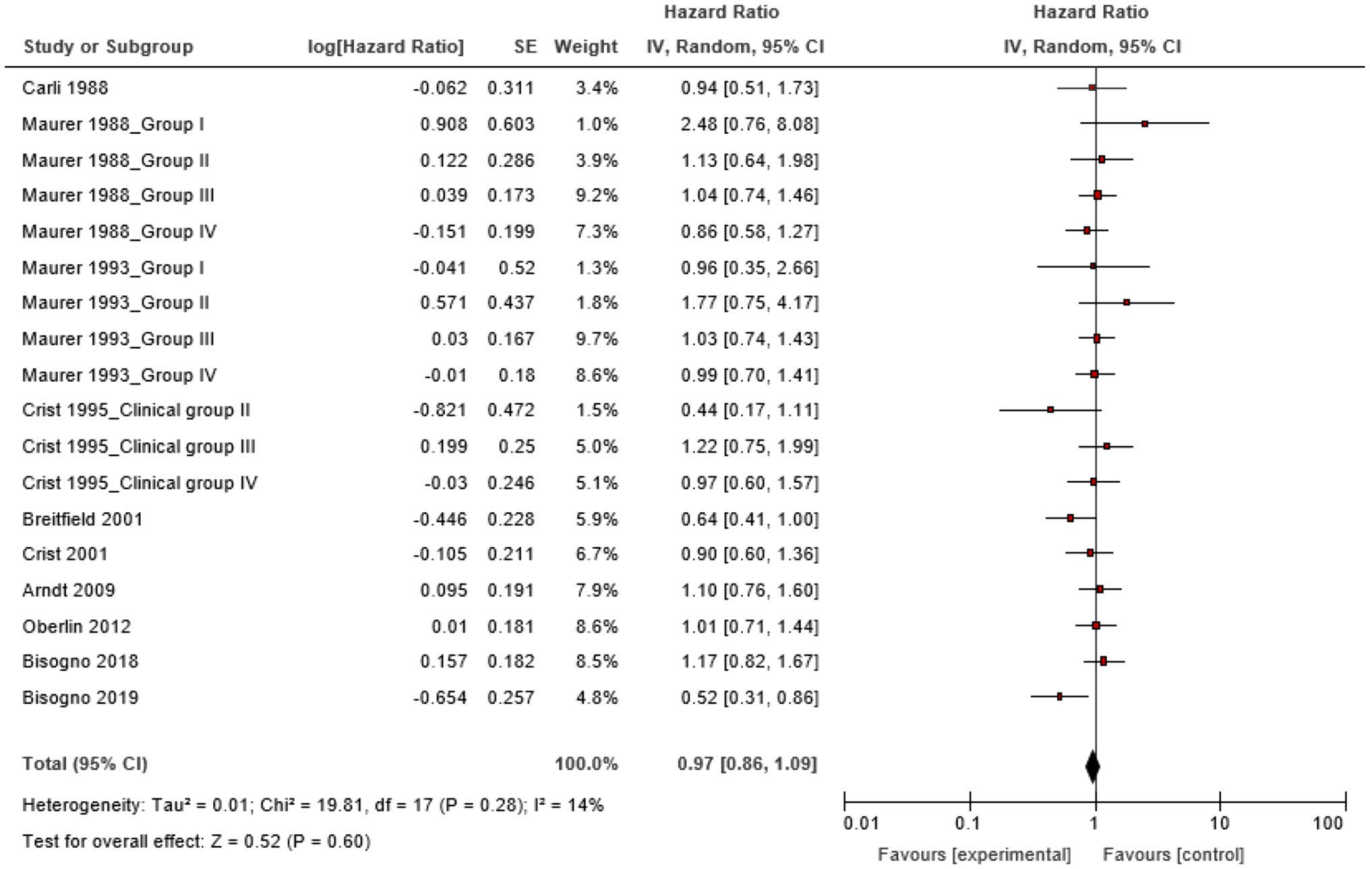
Forest plots of the hazard ratios (HR) for the overall survival (OS) and 95% confidence interval (CI) according to treatment effect sizes.

We performed a meta-analysis of OS HR using the random-effect model and found that the experimental arms failed to differ significantly from the control arms (HR 0.97, 95% CI 0.86–1.09, *P* = 0.60) (Fig. 2). This study showed no evidence of statistical heterogeneity in OS HR (*P* = 0.28; I^2^ = 14%) (Fig. 2). There was also no publication bias from the funnel plot (Supplementary Fig. 1). In contrast, the experimental arms were significantly more favorable than the control arms in terms of 1-year EFS (OR 0.76, 95% CI 0.63–0.91; *P* = 0.003) (Table 2, Supplementary Fig. 2) and RRs (CR + PR) (OR 1.35, 95% CI 1.03–1.77; *P* = 0.03) (Table 2, Supplementary Fig. 3). The control arms were significantly more favorable than the experimental arms in terms of overall severe adverse events (OR 1.55, 95% CI 1.20–1.99; *P* = 0.0007) (Table 2, Supplementary Fig. 4). The former showed a slight publication bias, but the latter two showed no publication bias in the funnel plots (Supplementary Figs. 5–7). The remaining intermediate end-points, ORs for 3- and 5-year EFS and 1-, 3-, and 5-year OS, and HR for EFS, did not show significant differences between the control and experimental arms (Table 2, Supplementary Figs. 8–13).

**Table 2:** Results of meta-analysis of treatment effect comparing the experimental arms with control arms.

End-point	HR/OR (95% CI)	*P*-value	No. of study arms
EFS	0.98 (0.88, 1.09)	0.74	14
1-year EFS	0.76 (0.63, 0.91)	0.003	14
3-year EFS	1.00 (0.87, 1.15)	0.97	14
5-year EFS	0.99 (0.84, 1.16)	0.87	14
OS	0.97 (0.86, 1.09)	0.60	18
1-year OS	0.82 (0.66, 1.01)	0.07	16
3-year OS	0.90 (0.76, 1.06)	0.21	18
5-year OS	0.98 (0.82, 1.16)	0.79	17
RR	1.35 (1.03, 1.77)	0.03	7
AEs, overall	1.55 (1.20, 1.99)	0.0007	7

Abbreviations: AE, adverse event; CI, confidence interval; EFS, event-free survival; HR, hazard ratio; OR, odds ratio; OS, overall survival; RR, response rate.

### Correlations between EFS and OS

**Figure 3:**
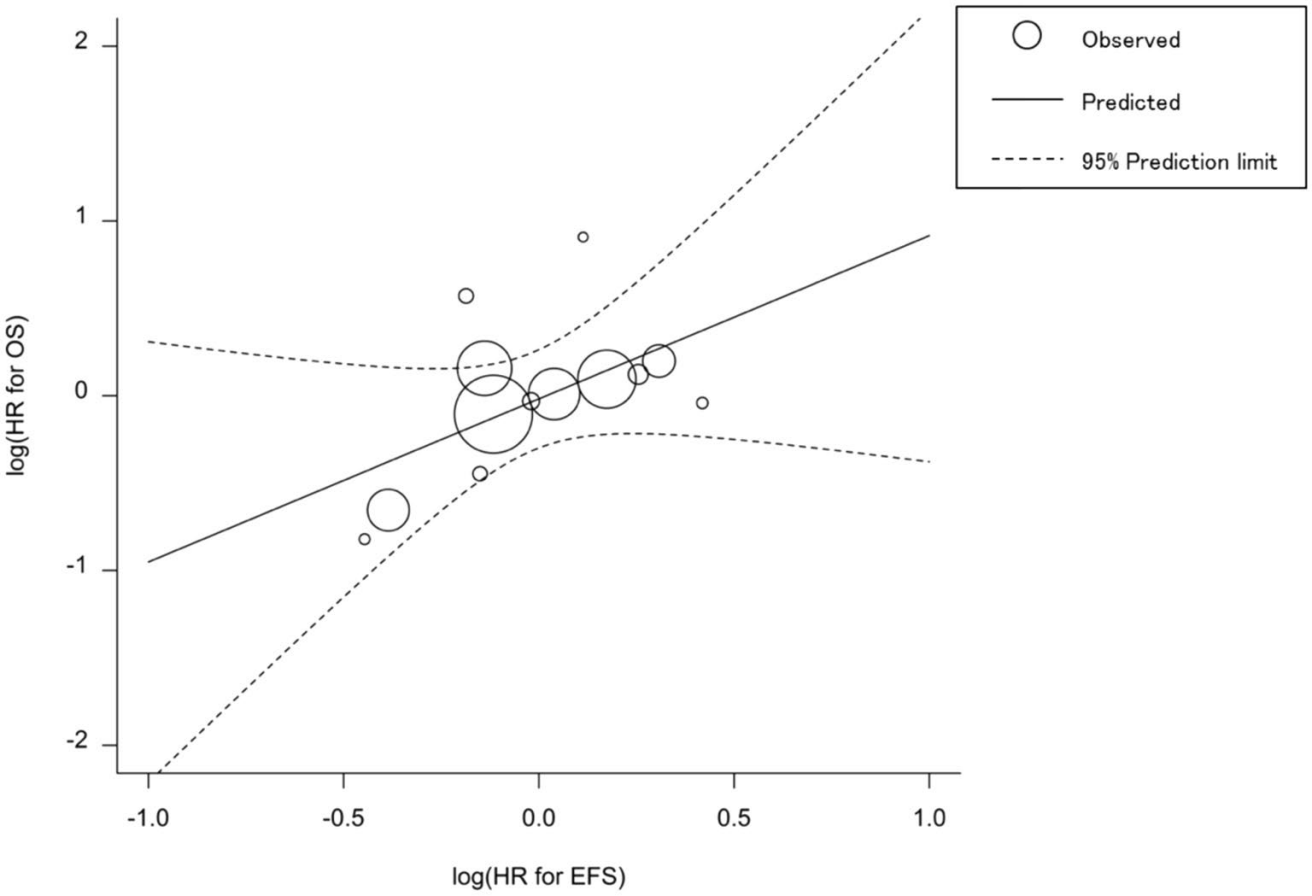
The trial-level association between the HR for event-free survival (EFS) and overall survival (OS).

The HR for EFS was moderately correlated to the HR for OS (Spearman’s rho = 0.445, 95% CI − 0.140 to 0.800, P = 0.1275). The equation for the regression line (Fig. 3) is as follows:

1 $${\text{log}}\left( {{\text{HR}}_{{{\text{OS}}}} } \right) = - 0.0{17} + 0.{934} \times {\text{log}}\left( {{\text{HR}}_{{{\text{EFS}}}} } \right)\,\left( {{95}\% {\text{ CI}} - 0.{312}\,{\text{to}}\,{2}.{18}0} \right)$$

This equation indicated that the risk reduction of OS HR was approximately 6.6% smaller than that of EFS HR. The trial-level R^2^ was 0.281 (95% CI 0.000–0.637), indicating slight association (Table 3, Fig. 3). Considering the 1-, 3-, 5-year EFS, the 3-year EFS had the strongest correlation with OS HR (R^2^ = 0.444; 95% CI 0.098–0.790; ρ = 0.593; 95% CI 0.063–0.862; *P* = 0.0325) (Table 3). Nevertheless, among the 1-, 3-, 5-year OS, the 3-year OS was well correlated to OS HR (R^2^ = 0.727, 95% CI 0.535–0.919; ρ = 0.781, 95% CI 0.495–0.915, *P* = 0.0001) and the 5-year OS was excellently correlated to OS HR (R^2^ = 0.911, 95% CI 0.839–0.983; ρ = 0.953, 95% CI 0.873–0.983, *P* < 0.0001) (Table 3). The other correlations were moderate or poor (Table 3).

**Table 3:** Results of surrogacy analysis of intermediate end-points for OS HR.

Surrogate end-point	R^2^ (95% CI)	ρ (95% CI)	*P*-value for ρ	No. of study arms
EFS	0.281 (0.000, 0.637)	0.445 (− 0.140, 0.800)	0.1275	13
1-year EFS	0.072 (0.000, 0.304)	0.313 (− 0.287, 0.737)	0.2974	13
3-year EFS	0.444 (0.098, 0.790)	0.593 (0.063, 0.862)	0.0325	13
5-year EFS	0.269 (0.000, 0.623)	0.478 (− 0.099, 0.814)	0.0985	13
1-year OS	0.012 (0.000, 0.108)	0.198 (− 0.349, 0.645)	0.4784	15
3-year OS	0.727 (0.535, 0.919)	0.781 (0.495, 0.915)	0.0001	17
5-year OS	0.911 (0.839, 0.983)	0.953 (0.873, 0.983)	< 0.0001	17

Abbreviations: CI, confidence interval; EFS, event-free survival; HR, hazard ratio; OS, overall survival.

### Sensitivity analysis

Subsequently, we performed a sensitivity analysis, excluding the treatment arms of the metastatic populations (Tables 4, 5). After excluding seven treatment arms (Carli 1988, Maurer 1988_Group IV, Maurer 1993_Group IV, Crist 1995_Clinical group IV, Breitfield 2001, Arndt 2009, Oberlin 2012), the correlation between the EFS HR and OS HR became poorer (R^2^ = 0.267, 95% CI 0.000–0.671; ρ = 0.400, 95% CI − 0.360 to 0.841, *P* = 0.2861) than that of the primary analysis (Table 5). The correlation between the EFS at 1, 3, and 5 years to OS HR deteriorated, whereas that between OS at 1, 3, and 5 years and OS HR improved (Table 5).

**Table 4:** Sensitivity analysis in meta-analysis of treatment effect comparing the experimental arms with control arms.

End-point	HR/OR (95% CI)	*P*-value	No. of studies
EFS	0.96 (0.83, 1.11)	0.57	10
1-year EFS	0.82(0.66, 1.02)	0.08	10
3-year EFS	0.97 (0.82, 1.15)	0.76	10
5-year EFS	0.96 (0.78, 1.18)	0.70	10
OS	1.00 (0.83, 1.22)	0.99	11
1-year OS	0.90 (0.67, 1.22)	0.51	10
3-year OS	0.93 (0.76, 1.14)	0.48	11
5-year OS	1.03 (0.78, 1.36)	0.84	10
RR	1.47 (1.03, 2.10)	0.04	3
AEs, overall	1.39 (0.96, 2.01)	0.09	5

Abbreviations: AE, adverse event; CI, confidence interval; EFS, event-free survival; HR, hazard ratio; OR, odds ratio; OS, overall survival; RR, response rate.

**Table 5:** Sensitivity analysis to evaluate surrogacy.

Surrogate end-point	R^2^ (95% CI)	ρ (95% CI)	*P*-value for ρ	No. of study arms
EFS	0.267 (0.000, 0.671)	0.400 (− 0.360, 0.841)	0.2861	9
1-year EFS	0.040 (0.000, 0.247)	0.233 (− 0.510, 0.777)	0.5457	9
3-year EFS	0.416 (0.013, 0.818)	0.617 (− 0.080, 0.909)	0.0769	9
5-year EFS	0.208 (0.000, 0.594)	0.400(− 0.360, 0.841)	0.2861	9
1-year OS	0.075 (0.000, 0.346)	− 0.092 (− 0.713, 0.609)	0.8138	9
3-year OS	0.826 (0.669, 0.983)	0.945 (0.798, 0.986)	< 0.0001	11
5-year OS	0.981 (0.961, 1.000)	0.952 (0.803, 0.989)	< 0.0001	10

Abbreviations: CI, confidence interval; EFS, event-free survival; OS, overall survival.

## Discussion

In RCTs, EFS, defined as the primary end-point, has some advantages over OS, as it requires a small sample size, shorter follow-up period for assessment, and low cost^12,21^. Moreover, EFS is not generally affected by post-progression treatments. Nonetheless, there is a disadvantage of being affected by the method used to evaluate and ascertain bias, such as knowledge of the therapy received^22^. It is possible that new diagnostic methods or anti-tumoral agents would dilute the surrogacy relationship in the future, even if the present surrogacy is reportedly strong^23^. Some reasons for the expectation of EFS as a surrogate end-point for OS are the social demand for early approval of new drugs and the theory that EFS is not only a surrogate end-point for OS but also makes positive contributions to the patients’ quality of life^21^. Although many surrogate end-point analyses have evaluated the EFS surrogacy for OS, a true end-point in RCTs for various malignant tumors, there has been no such study for RMS. This is the first report about surrogate end-point analyses for RMS and surrogacy analysis of intermediate end-points for OS.

The control arms of the studies were administered several cytotoxic agents, including vincristine and actinomycin, whereas the experimental arms received more toxic and multi-disciplinary treatments, additional radiotherapy, or anti-tumor agents. In the meta-analysis, the experimental arms were more favorable than the control arms in terms of 1-year EFS. However, longer endpoints such as 3- and 5-year EFS and 3- and 5-year OS did not show the superiority of experimental arms in RCTs conducted from 1974 were gathered. In other words, for EFS at a very early point, the experimental arms can be more favorable than the control arms, but for EFS at a later point, treatment effect between the arms would diminish. None of the new drug treatments have been highly successful. Overall, for RMS, there was only one RCT included in Table 1 showing that OS of the experimental arm was superior to that of the control arm. It is difficult to conduct clinical trials successfully due to a lack of effective drugs.

There are distinct prognoses and clinical courses between patients with localized RMS and those with metastatic RMS, even though these two groups of patients usually receive chemotherapy based on VAC. Therefore, we performed a sensitivity analysis excluding patients with metastatic RMS. After excluding metastatic patients with RMS, the HR of EFS was more poorly correlated with the HR of OS in the sensitivity analysis than in the primary analysis (Table 5). Likewise, the correlation between EFS at 1, 3, and 5 years and OS HR became progressively poor. In contrast, the correlation between 1-, 3-, and 5-year OS and OS HR improved accordingly. This result is similar to that previously reported for osteosarcoma^12^ and Ewing sarcoma^24^.

In general, it is considered that the longer the survival term following recurrence, the weaker the surrogacy of DFS for OS^25^. Broglio et al.^11^ reported that if there was a little difference in SPP between the two treatment arms, longer periods of SPP will weaken the association between PFS HR and OS HR and make it difficult to show a statistically significant difference in the OS between the treatment arms. Zer et al.^26^ evaluated published RCTs on locally advanced/metastatic STS, with systemic therapy in at least one arm. They finally identified 52 RCTs as eligible for analysis, including phase II and III studies, as in this study^26^. There were some differences from our study, that is, they included different treatment lines and various soft tissue sarcomas, and allowed control arms such as placebo or best supportive care^26^. The statistical analysis of the association between two endpoints was assessed using linear regression weighted by study sample size and association strength was assessed using the standardized β coefficient, not using coefficient of determination R^2^^26^. Their result showed significant correlations between PFS HR and OS HR and between RR OR and OS HR. The authors considered that shorter SPP, about 12 months, accounted for a large portion of eligible studies, which likely led to significant correlations between PFS HR and OS HR^26^. In contrast, as recommended by Schürmann et al.^20^, we utilized random effects meta-regression. The study mainly included patients with ERMS, who usually present longer SPP than those with other soft tissue sarcomas because the 5-year survival rate of patients with ERMS after relapse is 20–52%^27^. For this reason, the surrogacy of EFS for OS HR might have become weak in this study.

The relapse rate in patients with RMS who achieved complete remission or stable mass was 31.1–36.2%^28,29^. Additionally, recurrence occurred within 18 months after the first diagnosis in 50.4%–67.5% of relapsed patients^28,29^ and within 5 years after first diagnosis in 95% of relapsed patients^28^. The 5-year post-relapse survival rates in patients with group I and II/III ERMS were 52% and 20%, respectively^27^. However, the 5-year post-relapse survival rates in patients with groups I and II–IV ARMS or undifferentiated sarcoma were 40% and 3%, respectively^27^. In addition, the time to relapse after the end of the primary treatment has a significant influence on prognosis. Four-year survival rates after relapse within 6, 6–12, and more than 12 months were 12%, 21%, and 41%, respectively^30^. According to these results, ERMS with post-relapse survival was dominant because 11 RCTs used in this study comprised 53.9% of ERMS and 29.6% of ARMS; therefore, surrogacy of EFS for OS HR turned out to be weak. The 5-year EFS was more weakly correlated to OS HR than 3-year EFS, possibly because patients with early relapse RMS tend to die early and patients with late relapse patients tend to have long post-relapse survival. Another reason is that individual post-relapse treatments were heterogeneous. Some relapse patients may be able to undergo complete surgical excision and have long-term post-relapse survival^29^.

DFS has still not been validated to be a surrogate measure of OS in patients with breast cancer. Nevertheless, when patients with HER2-positive early breast cancer are selected as the object, DFS could be an acceptable surrogate for OS ^31^. Goldberg et al. ^22^ suggested that an appropriate biomarker, clinically defined patient selection, and receiving effective treatment would likely lead to restoration of PFS surrogacy for OS. In the case of RMS, which is known to be an ultra-rare sarcoma^32^, it seemed difficult to apply patient selection and specific treatment for RCTs of RMS. Thus, in terms of malignant tumors, it is important to discover or develop new biomarkers that can be acceptable surrogates for OS.

Our study has several limitations. First, we did not use individual data but published data. Second, most of the eligible RCTs did not describe the study phase. Third, only two RCTs described the ITT analysis. Finally, there were differences between each follow-up examination or later RCT treatment because eligible patients were registered for a long period (1979–2016).

This study concludes that when EFS is regarded as the primary end-point for an RCT of RMS, at least a three-year follow-up period is needed. Additionally, this result shows that the association between EFS and OS was modest, and EFS surrogacy for OS in RCTs of RMS was not confirmed. Therefore, it is necessary to discover or develop new biomarkers for RMS that can be an acceptable surrogate for OS.

## Supplementary Information

Supplementary Information.

## Data Availability

The datasets generated and/or analyzed in the current study are available from the corresponding author upon reasonable request.
