# Supplementary material for: Surrogacy analysis of intermediate end-points for overall survival in randomized controlled trials of rhabdomyosarcoma
Source: Sci Rep. 2022 Nov 12;12:19381. doi: 10.1038/s41598-022-23944-w (PMC9653385; doi:10.1038/s41598-022-23944-w)
Supplement: Supplementary file 1 — Supplementary Information. [file 41598_2022_23944_MOESM1_ESM.pptx]

## Slide 1
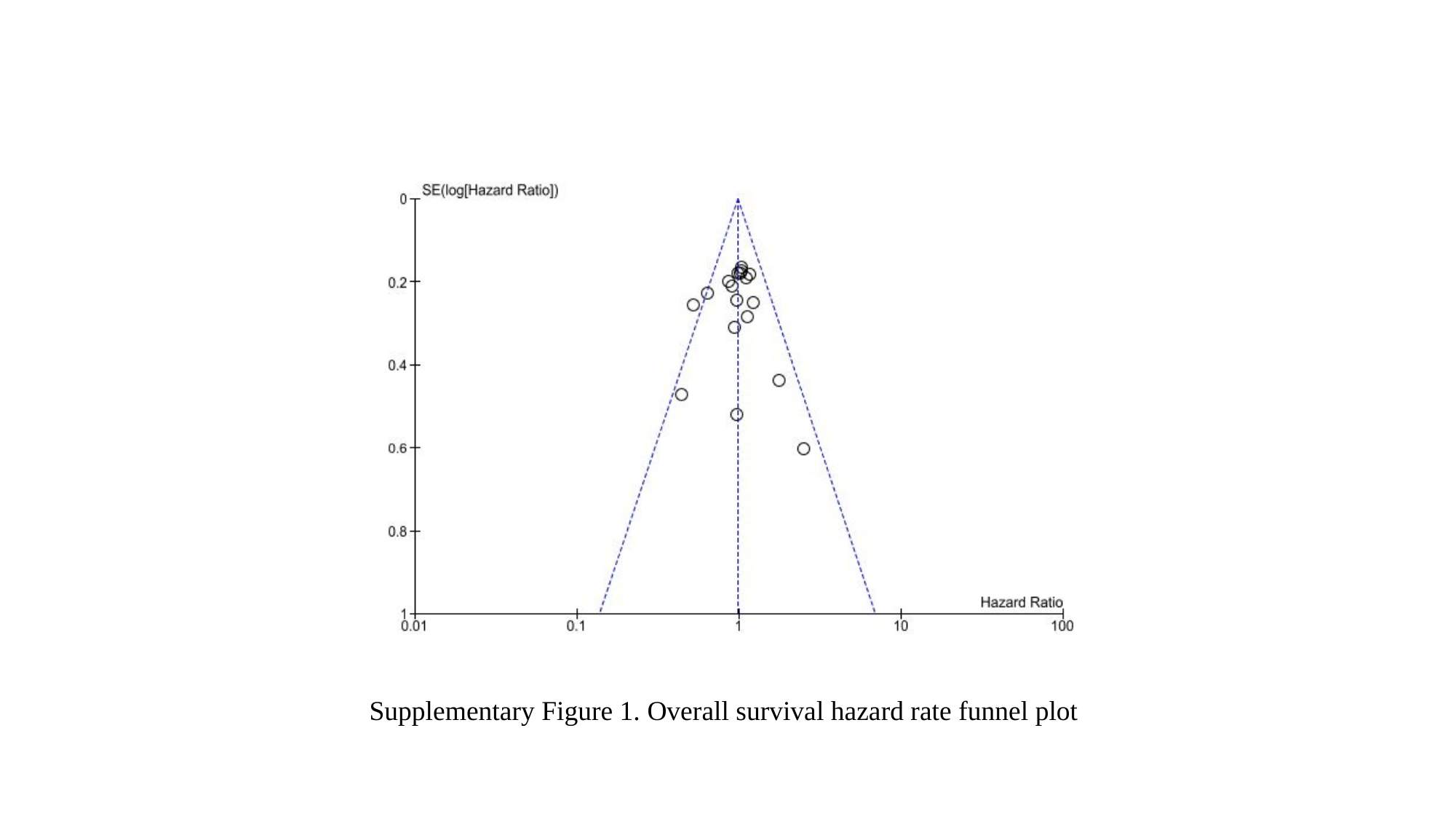

Supplementary Figure 1. Overall survival hazard rate funnel plot

## Slide 2
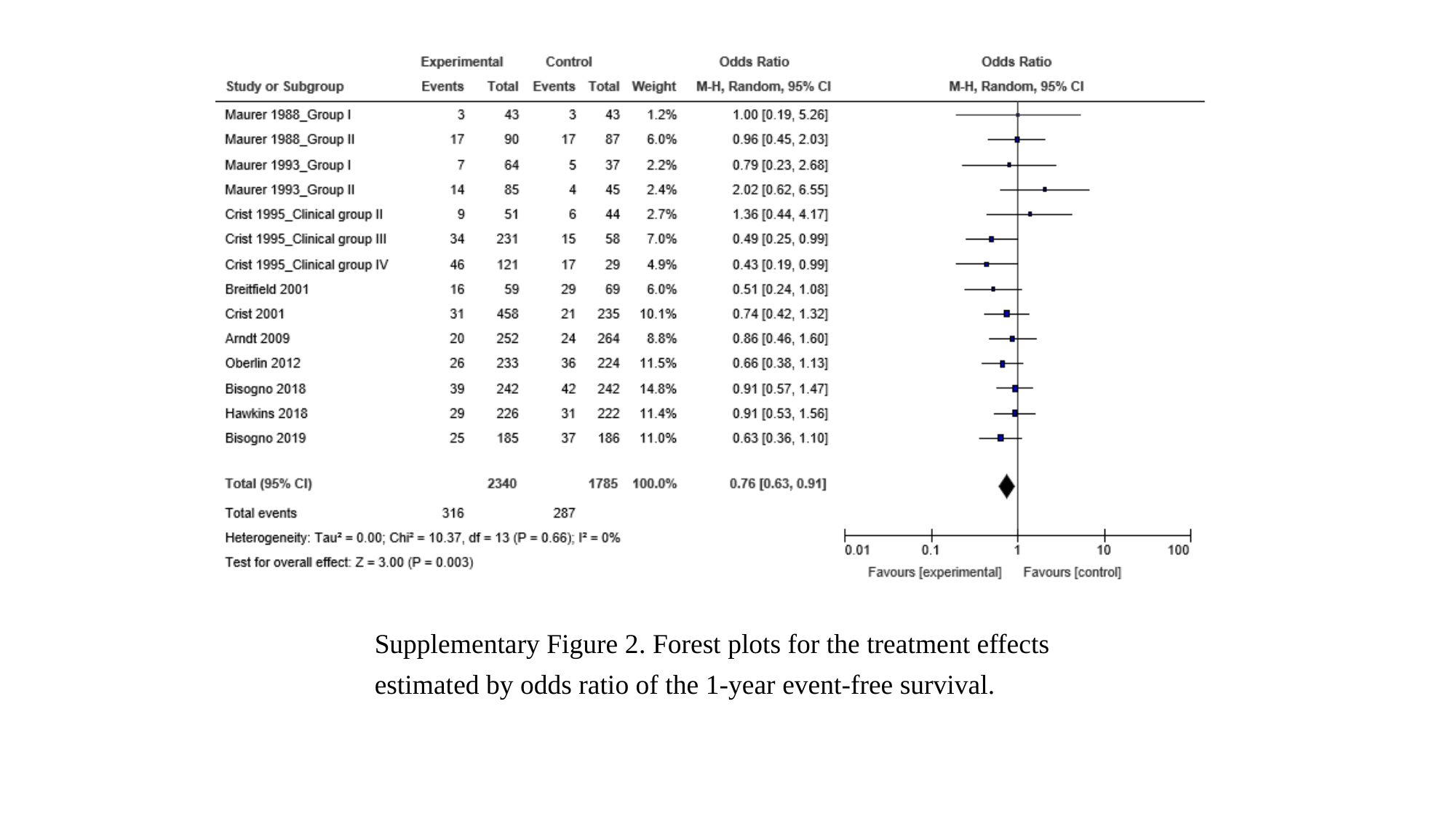

Supplementary Figure 2. Forest plots for the treatment effects estimated by odds ratio of the 1-year event-free survival.

## Slide 3
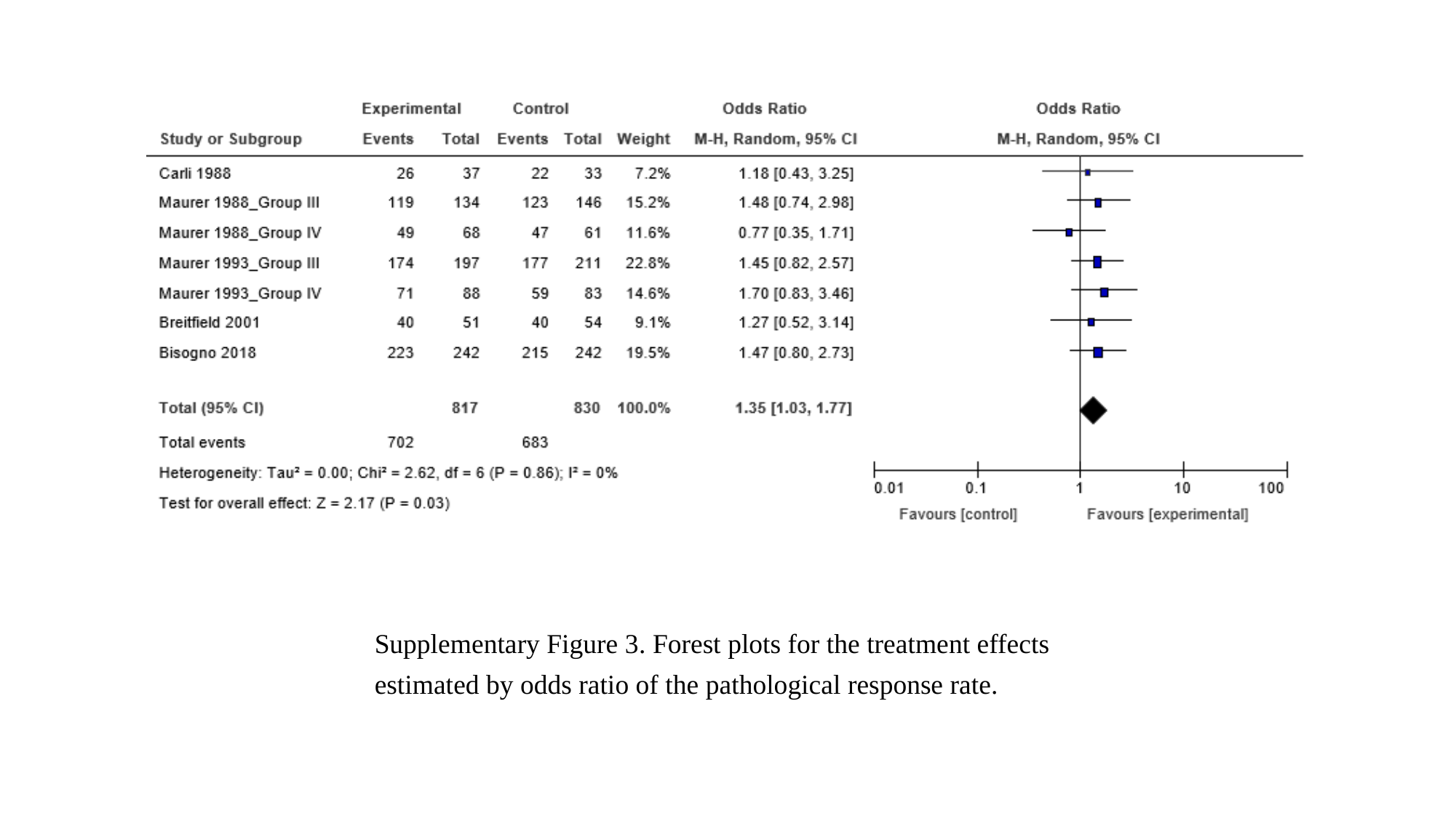

Supplementary Figure 3. Forest plots for the treatment effects estimated by odds ratio of the pathological response rate.

## Slide 4
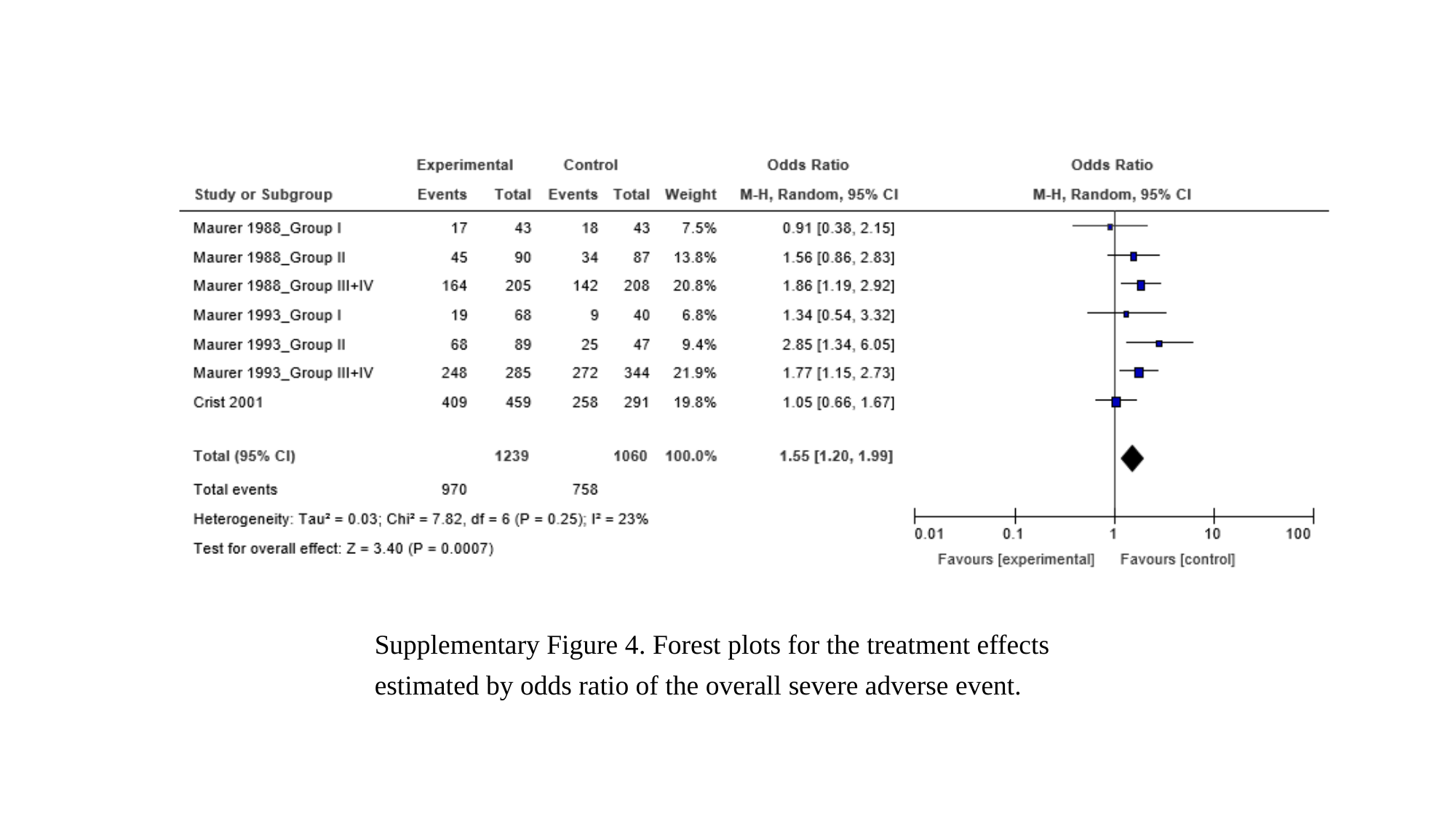

Supplementary Figure 4. Forest plots for the treatment effects estimated by odds ratio of the overall severe adverse event.

## Slide 5
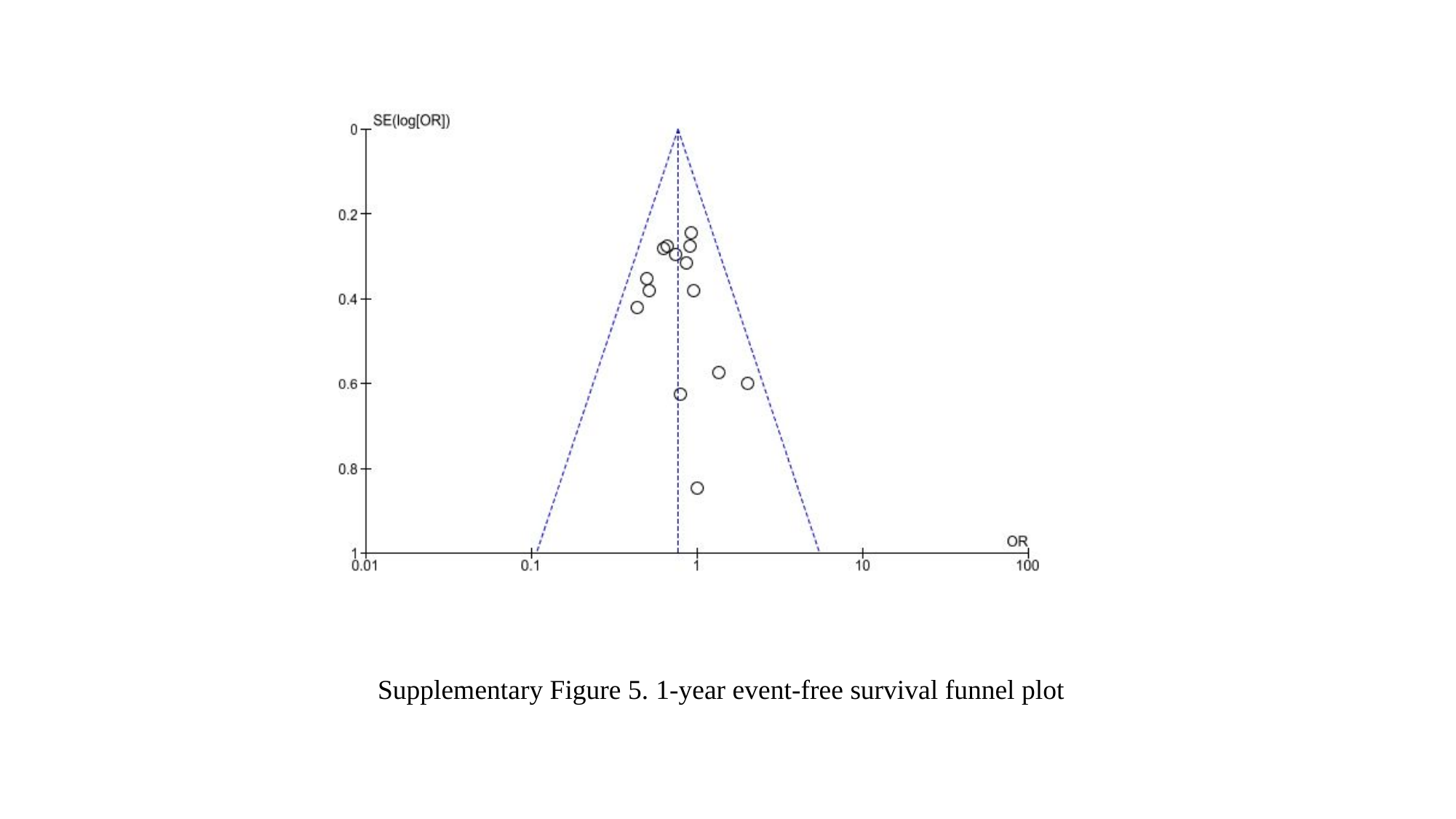

Supplementary Figure 5. 1-year event-free survival funnel plot

## Slide 6
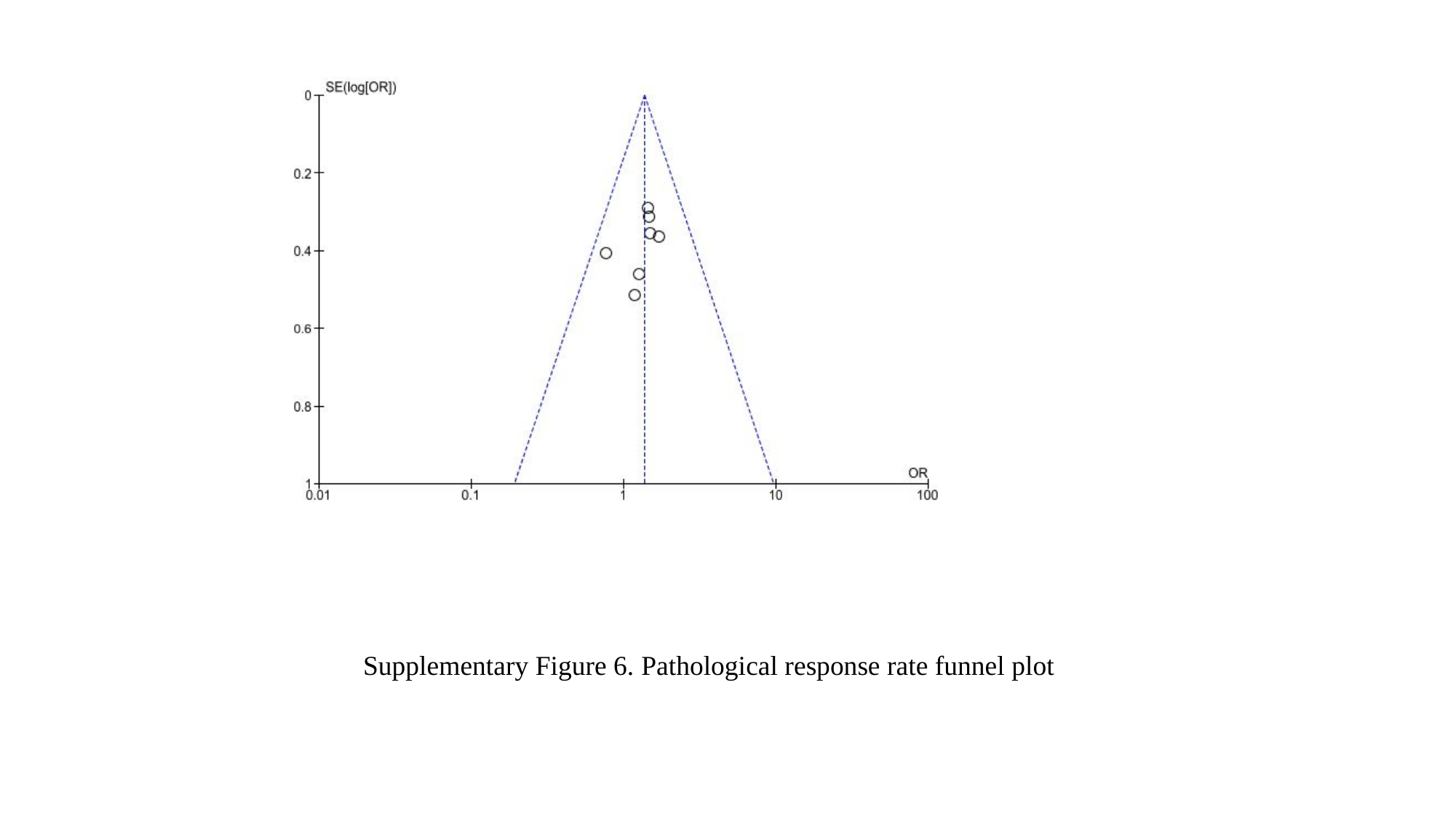

Supplementary Figure 6. Pathological response rate funnel plot

## Slide 7
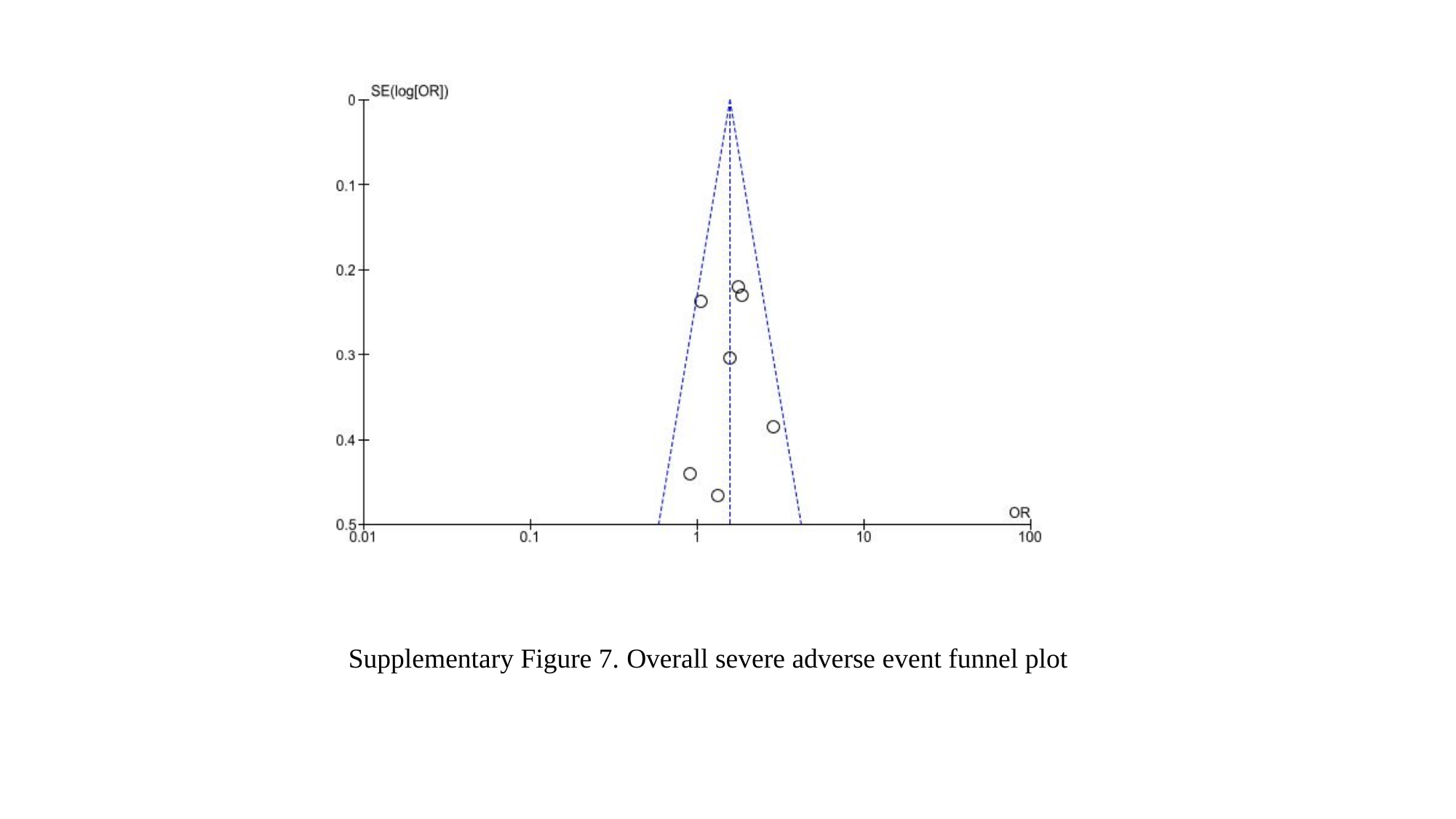

Supplementary Figure 7. Overall severe adverse event funnel plot

## Slide 8
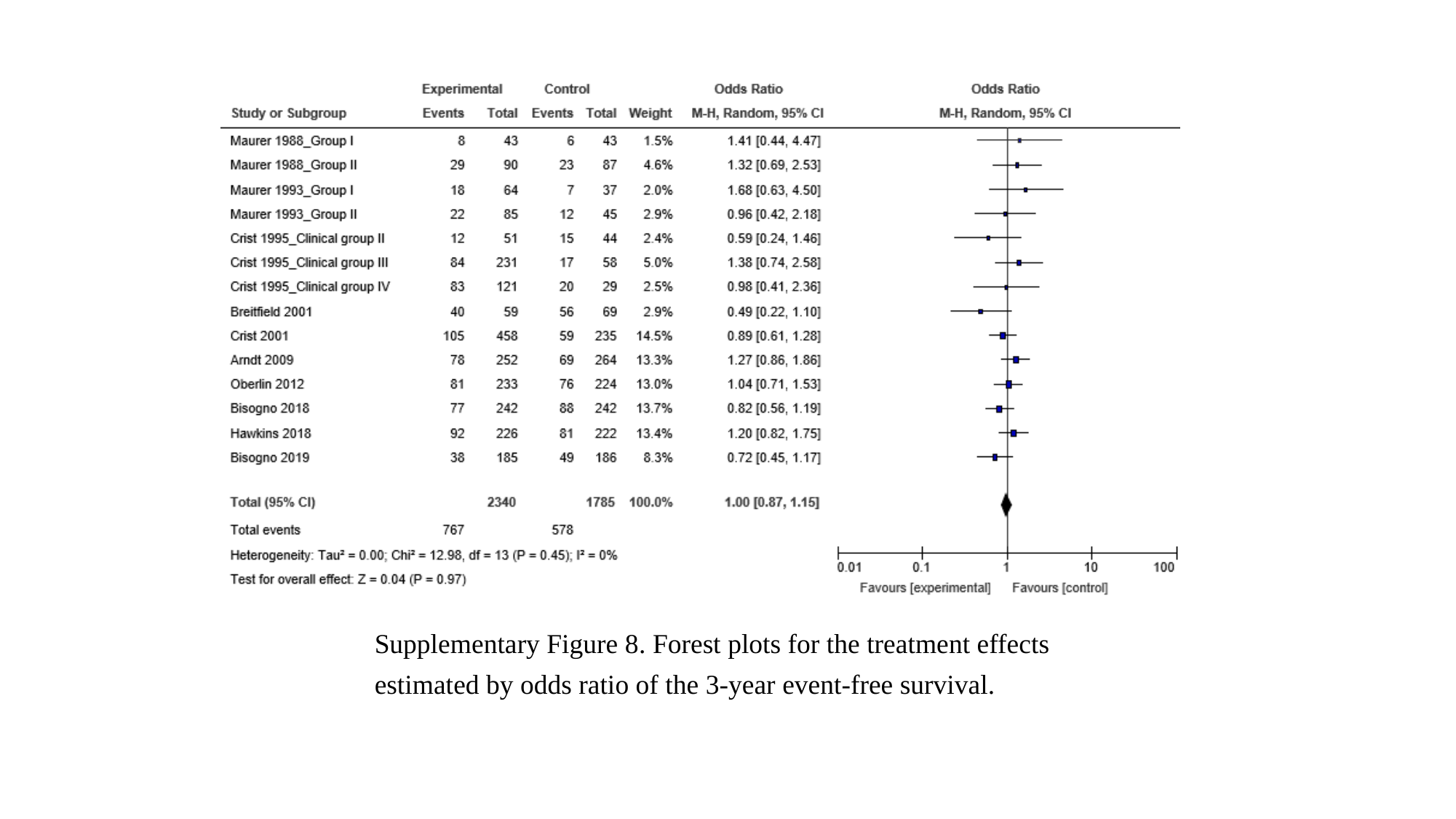

Supplementary Figure 8. Forest plots for the treatment effects estimated by odds ratio of the 3-year event-free survival.

## Slide 9
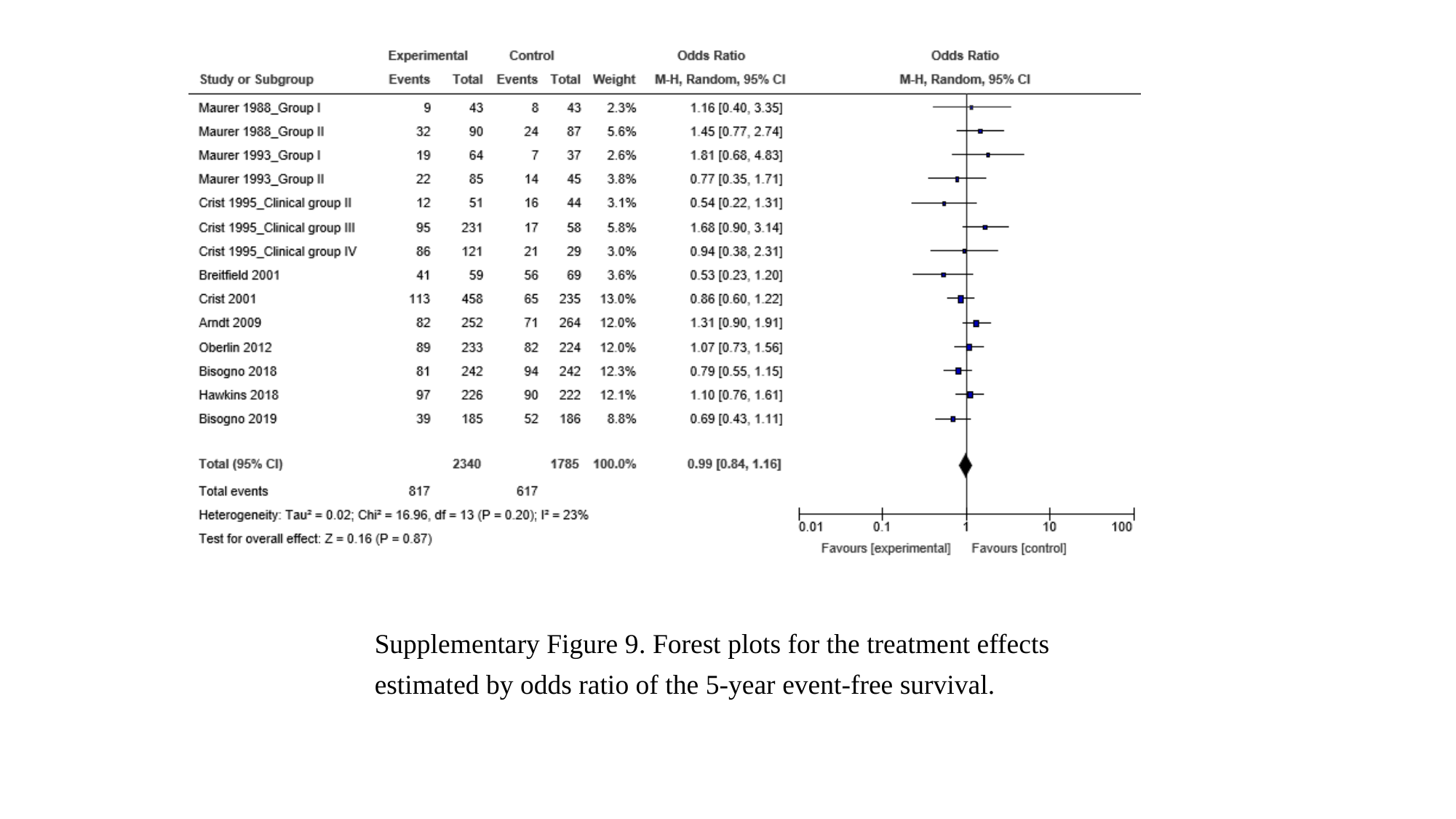

Supplementary Figure 9. Forest plots for the treatment effects estimated by odds ratio of the 5-year event-free survival.

## Slide 10
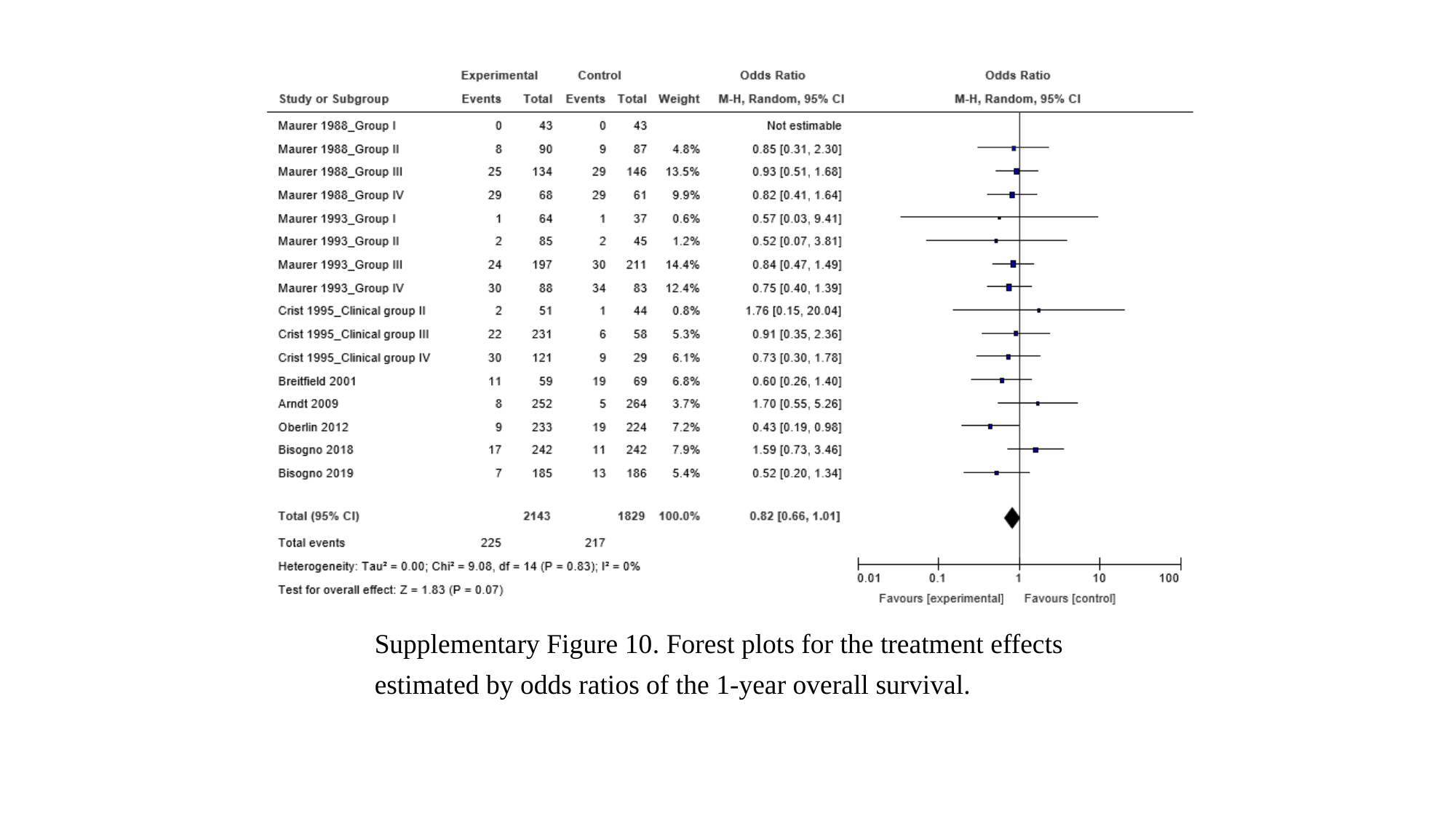

Supplementary Figure 10. Forest plots for the treatment effects estimated by odds ratios of the 1-year overall survival.

## Slide 11
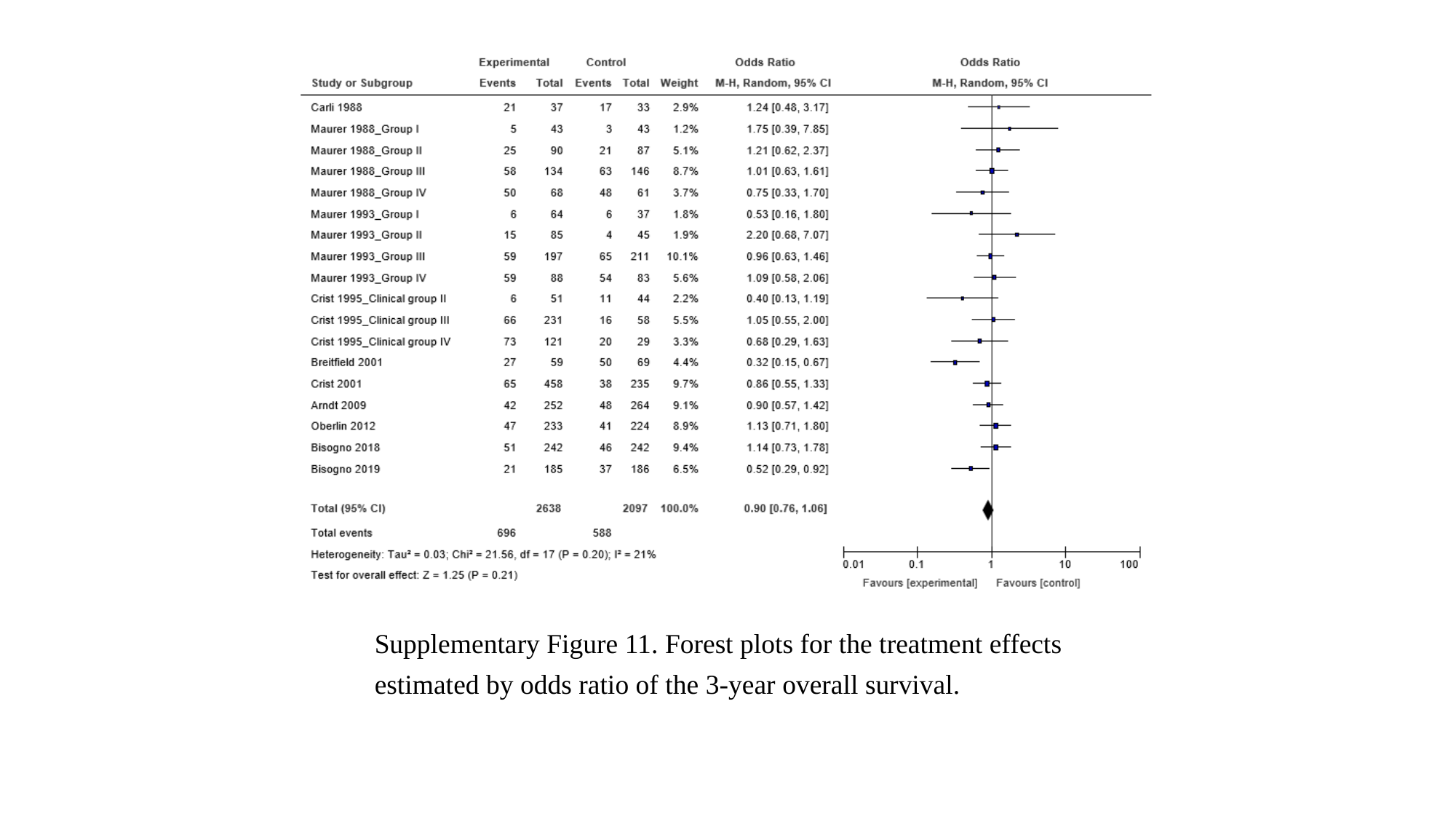

Supplementary Figure 11. Forest plots for the treatment effects estimated by odds ratio of the 3-year overall survival.

## Slide 12
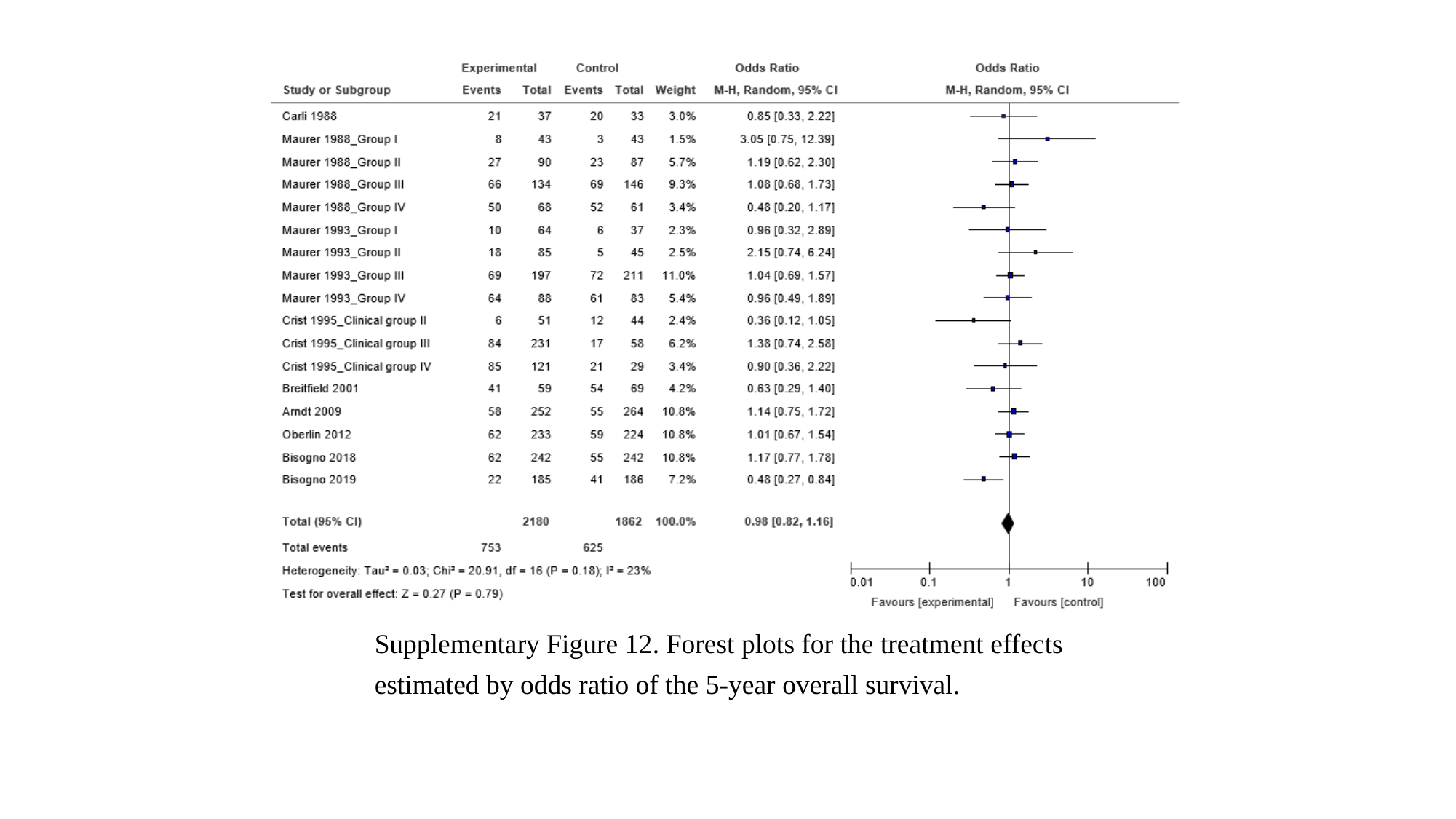

Supplementary Figure 12. Forest plots for the treatment effects estimated by odds ratio of the 5-year overall survival.

## Slide 13
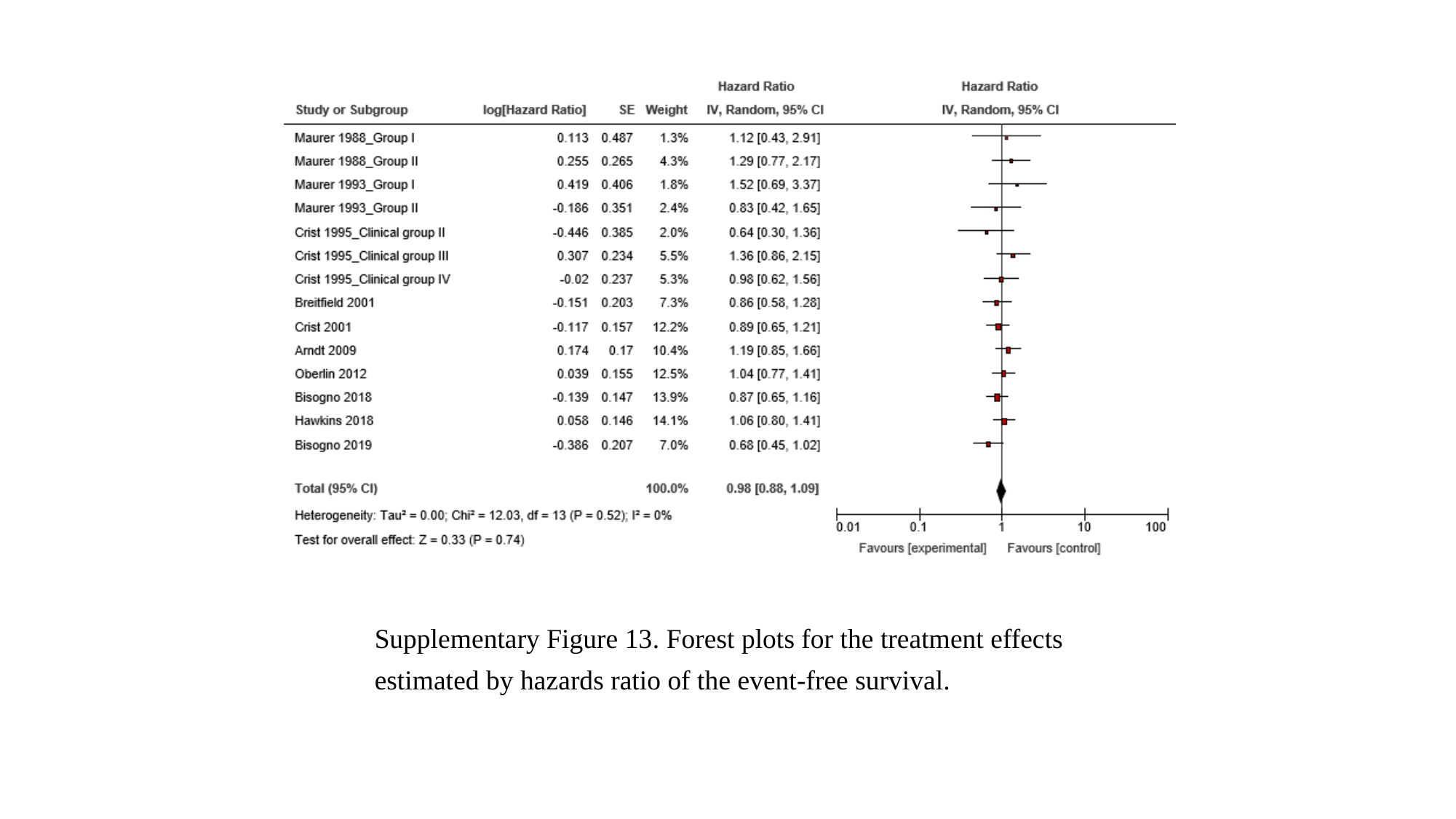

Supplementary Figure 13. Forest plots for the treatment effects estimated by hazards ratio of the event-free survival.
